# Ultrapotent antibodies against diverse and highly transmissible SARS-CoV-2 variants

**DOI:** 10.1126/science.abh1766

**Published:** 2021-08-13

**Authors:** Lingshu Wang, Tongqing Zhou, Yi Zhang, Eun Sung Yang, Chaim A. Schramm, Wei Shi, Amarendra Pegu, Olamide K. Oloniniyi, Amy R. Henry, Samuel Darko, Sandeep R. Narpala, Christian Hatcher, David R. Martinez, Yaroslav Tsybovsky, Emily Phung, Olubukola M. Abiona, Avan Antia, Evan M. Cale, Lauren A. Chang, Misook Choe, Kizzmekia S. Corbett, Rachel L. Davis, Anthony T. DiPiazza, Ingelise J. Gordon, Sabrina Helmold Hait, Tandile Hermanus, Prudence Kgagudi, Farida Laboune, Kwanyee Leung, Tracy Liu, Rosemarie D. Mason, Alexandra F. Nazzari, Laura Novik, Sarah O’Connell, Sijy O’Dell, Adam S. Olia, Stephen D. Schmidt, Tyler Stephens, Christopher D. Stringham, Chloe Adrienna Talana, I-Ting Teng, Danielle A. Wagner, Alicia T. Widge, Baoshan Zhang, Mario Roederer, Julie E. Ledgerwood, Tracy J. Ruckwardt, Martin R. Gaudinski, Penny L. Moore, Nicole A. Doria-Rose, Ralph S. Baric, Barney S. Graham, Adrian B. McDermott, Daniel C. Douek, Peter D. Kwong, John R. Mascola, Nancy J. Sullivan, John Misasi

**Affiliations:** 1 Vaccine Research Center, National Institute of Allergy and Infectious Diseases, National Institutes of Health, Bethesda, MD 20892, USA.; 2 Department of Epidemiology, UNC Chapel Hill School of Public Health, University of North Carolina School of Medicine, Chapel Hill, NC 27599, USA.; 3 Department of Microbiology and Immunology, University of North Carolina School of Medicine, Chapel Hill, NC 27599, USA.; 4 Electron Microscopy Laboratory, Cancer Research Technology Program, Leidos Biomedical Research, Frederick National Laboratory for Cancer Research, Frederick, MD 21702, USA.; 5 National Institute for Communicable Diseases (NICD) of the National Health Laboratory Service (NHLS), Johannesburg, South Africa.; 6 SAMRC Antibody Immunity Research Unit, School of Pathology, Faculty of Health Sciences, University of the Witwatersrand, Johannesburg, South Africa.

## Abstract

The emergence of highly transmissible SARS-CoV-2 variants of concern (VOCs) that are resistant to therapeutic antibodies highlights the need for continuing discovery of broadly reactive antibodies. We identified four receptor binding domain–targeting antibodies from three early-outbreak convalescent donors with potent neutralizing activity against 23 variants, including the B.1.1.7, B.1.351, P.1, B.1.429, B.1.526, and B.1.617 VOCs. Two antibodies are ultrapotent, with subnanomolar neutralization titers [half-maximal inhibitory concentration (IC_50_) 0.3 to 11.1 nanograms per milliliter; IC_80_ 1.5 to 34.5 nanograms per milliliter). We define the structural and functional determinants of binding for all four VOC-targeting antibodies and show that combinations of two antibodies decrease the in vitro generation of escape mutants, suggesting their potential in mitigating resistance development.

## Defenses against SARS-CoV-2 variants

Our key defense against the COVID-19 pandemic is neutralizing antibodies against the SARS-CoV-2 virus elicited by natural infection or vaccination. Recent emerging viral variants have raised concern because of their potential to escape antibody neutralization. Wang *et al*. identified four antibodies from early-outbreak convalescent donors that are potent against 23 variants, including variants of concern, and characterized their binding to the spike protein of severe acute respiratory syndrome coronavirus 2 (SARS-CoV-2). Yuan *et al*. examined the impact of emerging mutations in the receptor-binding domain of the spike protein on binding to the host receptor ACE2 and to a range of antibodies. These studies may be helpful for developing more broadly effective vaccines and therapeutic antibodies. —VV

## Abstract (structured)

**INTRODUCTION:**

Worldwide appearance of severe acute respiratory syndrome coronavirus 2 (SARS-CoV-2) variants of concern (VOCs) with increased transmissibility and resistance to therapeutic antibodies necessitates the discovery of broadly reactive antibodies. We isolated receptor binding domain (RBD) targeting antibodies that potently neutralize 23 variants, including the B.1.1.7, B.1.351, P.1, B.1.429, B.1.526, and B.1.617 VOCs. Structural and functional studies revealed the molecular basis for antibody binding and showed that antibody combinations reduce the generation of escape mutants, suggesting a potential means to mitigate development of therapeutic resistance.

**RATIONALE:**

Investigation of antibody responses from convalescent subjects infected with the Washington-1 (WA-1) strain for reactivity against WA-1 and VOCs can inform improvements to vaccine design and therapeutics.

**RESULTS:**

Blood from 22 convalescent subjects who recovered from SARS-CoV-2 WA-1 infection was screened for neutralizing and binding activity, and four subjects with high reactivity against the WA-1 variant were selected for antibody isolation. SARS-CoV-2 spike (S)–reactive antibodies were identified through B cell sorting with S protein–based probes. WA-1 live-virus neutralization assays identified four RBD-targeting antibodies with high potency [half-maximal inhibitory concentration (IC_50_) 2.1 to 4.8 ng/ml], two of which were derived from the same IGHV1-58 germline but from different donors. Antigen-binding fragments (Fabs) of these antibodies exhibited nanomolar affinity to S (2.3 to 7.3 nM). Competition assays and electron microscopy indicated that two of the most potent antibodies blocked angiotensin-converting enzyme 2 (ACE2) and bound open conformation RBD, whereas the other two bound both up and down conformations of RBD and blocked ACE2 binding. Binding and lentivirus neutralization assays against 13 circulating VOCs or variants of interest—including B.1.1.7, B.1.351, B.1.427, B.1.429, B.1.526, P.1, P.2, B.1.617.1, and B.1.617.2—indicated that these antibodies were highly potent against VOCs despite being isolated from subjects infected with early ancestral SARS-CoV-2 viruses. Cryo-EM studies of the two most potent antibodies in complex with S revealed that these antibodies target a site of vulnerability on RBD but have minimal contacts with mutational hotspots, defining the structural basis for their high effectiveness against the emerging VOCs and further delineating an IGHV1-58 antibody supersite. To investigate potential mechanisms of escape, we applied antibody selection pressure to replication-competent vesicular stomatitis virus (rcVSV) expressing the WA-1 SARS-CoV-2 S (rcVSV-SARS2) and identified S mutations that conferred in vitro resistance. We evaluated these antibodies individually or in combinations for their capacity to prevent rcVSV-SARS2 escape and discovered that antibody combinations with complementary modes of recognition to the RBD lowered the risk of resistance.

**CONCLUSION:**

Our study demonstrates that convalescent subjects previously infected with ancestral variant SARS-CoV-2 produce antibodies that cross-neutralize emerging VOCs with high potency. Structural and functional analyses reveal that antibody breadth is mediated by targeting a site of vulnerability at the RBD tip offset from major mutational hotspots in VOCs. Selective boosting of immune responses targeting specific RBD epitopes, such as the sites defined by these antibodies, may induce breadth against current and future VOCs.

## Teaser

Potently neutralizing antibodies from convalescent donors from the first wave of SARS-CoV-2 neutralize recent variants of concern.

## Graphical abstract

**Figure:**
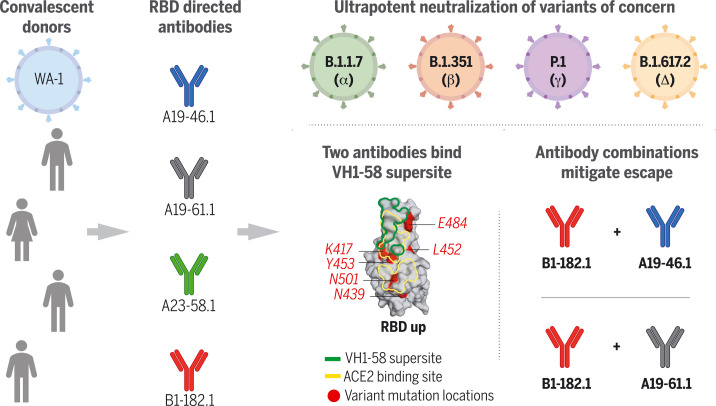
Isolation and characterization of convalescent donor antibodies that effectively neutralize emerging SARS-CoV-2 VOCs. Antibodies isolated from donors infected with ancestral SARS-CoV-2 viruses showed ultrapotent neutralization of emerging VOCs. The two most potent antibodies shared usage of the IGHV1-58 gene and targeted the RBD with minimal contact to VOC mutational hotspots. Cocktails of antibodies with complementary binding modes suppressed antibody escape.

Since the start of the severe acute respiratory syndrone coronavirus 2 (SARS-CoV-2) outbreak, >170 million people have been infected, and >3.7 million have died from COVID-19 (*1*). The virus is decorated with a trimeric spike protein (S), which comprises an S1 subunit that binds host cells and an S2 subunit that is responsible for membrane fusion. The S1 subunit comprises an N-terminal domain (NTD); the receptor binding domain (RBD) that binds the host angiotensin-converting enzyme 2 (ACE2) receptor; and two additional subdomains, SD1 and SD2. Shortly after the first Wuhan Hu-1 (Hu-1) genome sequence was published (*2*), S proteins based on this sequence were generated for use in antibody discovery (*3*–*5*). SARS-CoV-2 variants such as B.1.1.7 (for example, Alpha, 501Y.V1) (*6*), B.1.351 (for example, Beta, 501Y.V2) (*7*), P.1 (for example, Gamma, 501Y.V3), and B.1.617.2 (for example, Delta, 452R.V3) (*8*, *9*) contain mutations, many in S, that mediate resistance to therapeutic monoclonal antibodies, have increased transmissibility, and potentially increase pathogenicity (*10*–*14*). Vaccines designs based on the original Hu-1 outbreak strain sequence elicit antibody responses that show decreased in vitro neutralizing activity against variants (*14*–*16*). In this study, antibodies isolated from convalescent subjects who were infected by the Washington-1 (WA-1) strain, which has an identical S sequence to Hu-1, were investigated for reactivity against WA-1 and variants of concern (VOCs), and we defined the structural features of their binding to S.

## Identification and characterization of antibodies against WA-1

**Fig. 1.**
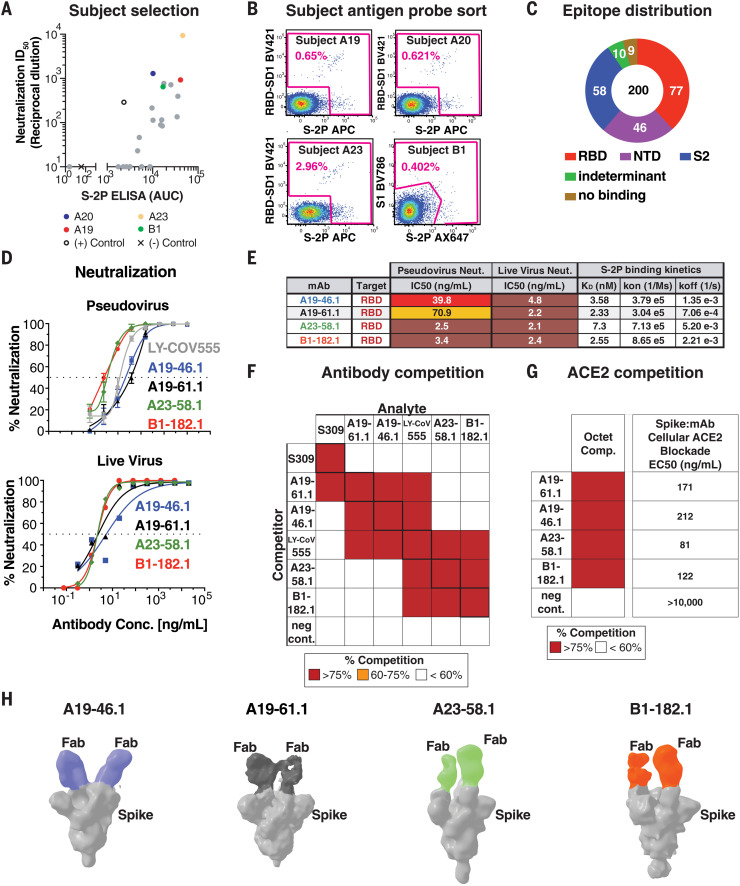
Identification and classification of highly potent antibodies from convalescent SARS-CoV-2 subjects. (**A**) Sera from 22 convalescent subjects were tested for neutralizing (*y* axis, ID_50_) and binding antibodies (*x* axis, S-2P ELISA AUC), and four subjects—A19, A20, A23, and B1 (colored) with both high neutralizing and binding activity against the WA-1—were selected for antibody isolation. (**B**) Final flow cytometry sorting gate of CD19^+^/CD20^+^/IgG^+^ or IgA^+^ PBMCs for four convalescent subjects (A19, A20, A23, and B1). Shown is the staining for RBD-SD1 BV421, S1 BV786, and S-2P APC or Ax647. Cells were sorted by using indicated sorting gate (pink), and percent of positive cells that were either RBD-SD1-, S1-, or S-2P-– positive is shown for each subject. (**C**) Gross binding epitope distribution was determined by using an MSD-based ELISA testing against RBD, NTD, S1, S-2P, or HexaPro. S2 binding was inferred from S-2P or HexaPro binding without binding to other antigens. Indeterminant epitopes showed a mixed binding profile. Total number of antibodies (200) and absolute number of antibodies within each group is shown. (**D**) Neutralization curves by using WA-1 spike pseudotyped lentivirus and live virus neutralization assays to test the neutralization capacity of the indicated antibodies (*n* = 2 to 3 replicates). (**E**) Table showing antibody binding target, IC_50_ for pseudovirus and live virus neutralization, and Fab:S-2P binding kinetics (*n* = 2 replicates) for the indicated antibodies. (**F**) SPR-based epitope binning experiment. Competitor antibody (*y* axis) is bound to S-2P before incubation with the analyte antibody (*x* axis) as indicated, and percent competition range bins are shown as red (>75%), orange (60 to 75%), or white (<60%) (*n* = 2 replicates). Negative control antibody is anti-Ebola glycoprotein antibody mAb114 (*37*). (**G**) Competition of ACE2 binding. The indicated antibodies (*y* axis) complete binding of S-2P to soluble ACE2 protein by using biolayer interferometry [left column, percent competition (>75% shown as red, <60% as white)] or to cell surface–expressed ACE2 by using cell-surface staining (right column, EC_50_ at ng/ml shown). (**H**) Negative-stain 3D reconstructions of SARS-CoV-2 spike and Fab complexes. A19-46.1 and A19-61.1 bind to RBD in the down position, whereas A23-58.1 and B1-182.1 bind to RBD in the up position. Representative classes were shown with two Fabs bound, although stoichiometry at one to three Fabs was observed.

We obtained blood from 22 convalescent subjects, who had experienced mild to moderate symptoms after WA-1 infection, between 25 and 55 days after symptom onset. Four subjects—A19, A20, A23, and B1—had both high neutralizing and binding activity against the WA-1 variant (Fig. 1A) and were selected for antibody isolation efforts. CD19^+^/CD20^+^/immunoglobulin M^–^ (IgM^–^)/IgA^+^ or IgG^+^ B cells were sorted for binding to a stabilized version of S (S-2P), the full S1 subunit, or the RBD plus the subdomain-1 region of S1 (RBD-SD1) (Fig. 1B and fig. S1). In total, we sorted 889 B cells, recovered 709 (80%) paired heavy- and light-chain antibody sequences, and selected 200 antibodies for expression. A meso scale discovery (MSD) binding assay was used to measure binding of these 200 antibodies to stabilized spike, the full S1 subunit, RBD, or NTD. There was a broad response across all spike domains with 77 binding RBD, 46 binding NTD, 58 inferred to bind the S2 subunit based on binding to S but not to S1, and 19 binding an indeterminant epitope or failing to recognize spike in an MSD binding assay (Fig. 1C).

Pseudovirus neutralization assays by using the WA-1 spike showed that four RBD targeting antibodies—A19-46.1, A19-61.1, A23-58.1, and B1-182.1 (table S1)—are especially potent [half-maximal inhibitory concentration (the concentration of an antibody required to inhibit virus entry by 50%) (IC_50_) 2.5 to 70.9 ng/ml] (Fig. 1, D and E). WA-1 live virus neutralization (*17*) revealed similar high potent neutralization by all four antibodies (IC_50_ 2.1 to 4.8 ng/ml) (Fig. 1, D and E). All four antibody Fabs exhibited nanomolar affinity for SARS-CoV-2 S-2P (2.3 to 7.3 nM), which is consistent with their potent neutralization (Fig. 1E).

Antibodies targeting the RBD can be categorized into four general classes (classes I to IV) on the basis of competition with the ACE2 target cell receptor protein for binding to S and recognition of the up or down state of the three RBDs in S (*18*). LY-CoV555 is a therapeutic antibody that binds RBD in both the up and down states, blocks ACE2 binding, and is categorized as class II. However, despite potent activity against WA-1, VOCs have been reported to contain mutations that confer resistance to LY-CoV555 (*14*, *19*, *20*) and similarly binding antibodies. We therefore examined whether the epitopes targeted by the four high-potency antibodies were distinct from LY-CoV555. We used a surface plasmon resonance (SPR)–based competition binding assay to compare the binding profile of these antibodies to LY-CoV555. Although LY-CoV555 competed with A19-46.1, A19-61.1, A23-58.1, and B1-182.1 (and vice versa), their overall competition profiles were not the same. A23-58.1 and B1-182.1 exhibit similar binding profiles, and A19-61.1 and A19-46.1 likewise display a shared competition binding profile in our SPR assay. However, the latter two antibodies can be distinguished from each other owing to A19-61.1 competition with the class III antibody S309 (Fig. 1F) (*21*), which binds an epitope in RBD that is accessible in the up or down position but does not compete with ACE2 binding (*18*).

To determine whether the antibodies block ACE2 binding, we used biolayer interferometry ACE2-competition and cell-surface binding assays to show that all four antibodies prevent the binding of ACE2 to spike (Fig. 1G and fig. S2). This suggests that A19-46.1, A23-58.1, and B1-182.1 neutralize infection by directly blocking the interaction of RBD with ACE2 and would be classified as either class I (ACE2 blocking, binding RBD up only) or II (ACE2 blocking, binding RBD up or down) RBD antibodies (*18*). A19-61.1 competition with S309 and ACE2 binding suggests that it binds at least partly outside of the ACE2 binding motif but may sterically block ACE2 binding similar to the class III antibody REGN10987. To refine the classification of these antibodies, we performed negative-stain three-dimensional (3D) reconstruction and found that A19-46.1 and A19-61.1 bound near one another with all RBDs in the down position (Fig. 1H), which is consistent with them being class II and class III antibodies, respectively. Similarly, A23-58.1 and B1-182.1 bound to overlapping regions when RBDs are in the up position, suggesting that they are class I antibodies.

## Antibody binding and neutralization against circulating variants

**Fig. 2.**
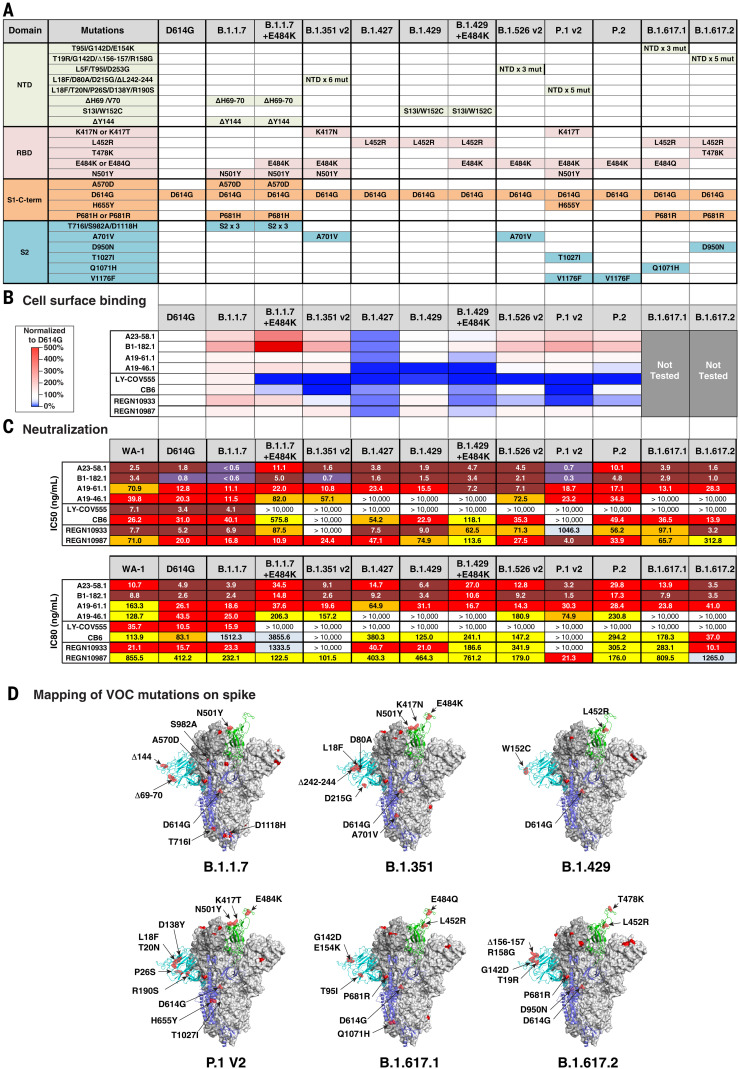
Antibody binding and neutralization of VOCs or VOIs. (**A**) Table showing domain and mutations relative to WA-1 for each of the 10 variants tested in (B) and (C). (**B**) Spike protein variants were expressed on the surface of HEK293 T cells, and binding to the indicated antibody was measured with flow cytometry. Data are shown as MFI normalized to the MFI for the same antibody against the D614G parental variant. Percent change is indicated by a color gradient from red (increased binding, Max 500%) to white (no change, 100%) to blue (no binding, 0%). (**C**) IC_50_ and IC_80_ values for the indicated antibodies against 10 variants shown in (A). Ranges are indicated with white (>10,000 ng/ml), light blue (>1000 to ≤10,000 ng/ml), yellow (>100 to ≤1000 ng/ml), orange (>50 to ≤100 ng/ml), red (>10 to ≤50 ng/ml), maroon (>1 to ≤10 ng/ml), and purple (≤1 ng/ml). (**D**) Location of spike protein variant mutations on the spike glycoprotein for B.1.1.7, B.1.351, B.1.429, P.1 v2, B.1.617.1, and B.1.617.2. P681 and V1176 are not resolved in the structure, and therefore their locations are not noted in B.1.1.7 and P.1 v2.

Because each donor subject was infected with a variant close to the ancestral WA-1, we evaluated antibody activity against recently emerged variants such as D614G, which has become the dominant variant across the world (*22*). Similar to LY-CoV555, neutralization potency was increased against D614G compared with WA-1, with the IC_50_ and IC_80_ of each experimental antibody 1.4- to 6.3-fold lower than that seen for the WA-1 (IC_50_ of 0.8 to 20.3 ng/ml and IC_80_ of 2.6 to 43.5 ng/ml) (Fig. 2, A and C, and fig. S3). [Single-letter abbreviations for the amino acid residues are as follows: A, Ala; C, Cys; D, Asp; E, Glu; F, Phe; G, Gly; H, His; I, Ile; K, Lys; L, Leu; M, Met; N, Asn; P, Pro; Q, Gln; R, Arg; S, Ser; T, Thr; V, Val; W, Trp; and Y, Tyr. In the mutants, other amino acids were substituted at certain locations; for example, D614G indicates that aspartic acid at position 614 was replaced by glycine.]

Next, we assessed antibody binding to D614G and nine additional cell surface–expressed spike variants that have appeared subsequent to WA-1 and that are not considered VOCs or variants of interest (VOIs) (B.1.1.7.14, B.1.258.24, Y453F/D614G, Ap.1, B.1.388, ΔH69-70/N501Y/D614G, K417N/D614G, B.1.1.345, and B.1.77.31) (*6*–*9*, *22*). Experimental antibodies were compared with four antibodies that are in clinical use [LY-CoV555, REGN10933, REGN10987, and CB6 (LY-CoV016)]. All control and experimental antibodies showed a minor reduction in binding (less than twofold) to B.1.258.24 (N439K/D614G) (figs. S3 and S4). Despite this, their neutralization capacities were minimally affected, with the exception of REGN10987 (2005 ng/ml) as reported previously (figs. S3 and S4) (*23*). Whereas none of the experimental antibodies showed large reductions in binding, LY-CoV555, CB6 (*24*), and REGN10933 (*25*) each showed >10-fold binding deficits to one or more variants (Y453F/D614G, K417N/D614G, B.1.1.345, or B.1.177.31) in these cell-based binding assays (figs. S3 and S4).

We next evaluated the capacity of each antibody to neutralize lentiviral particles pseudotyped with the same 10 variant spike proteins. Consistent with published data, REGN10933 did not neutralize Y453F/D614G or B.1.177.31 (K417N/E484K/N501Y/D614G) (*12*, *14*, *26*); CB6 did not neutralize B.1.177.31; and LY-CoV555 and REGN109333 showed potency reductions of 28- to >1400-fold for neutralization of viruses containing E484K (fig. S3) (*12*, *14*). Relative to WA-1, the A23-58.1 IC_50_ neutralization was threefold lower for ΔH69-70/N501Y/D614G (0.9 ng/ml) and fivefold lower for Ap.1 (<0.6 ng/ml), and although A23-58.1 maintained high potency, neutralization against B.1.1.345 was increased fourfold (10.2 ng/ml). Neutralization by B1-182.1 maintained high potency (IC_50_ < 3.2 ng/ml) for all variants and showed more than fourfold improved potency for 6 of the 10 variants tested (IC_50_ < 0.8 ng/ml) (fig. S3). For A19-61.1, variant neutralization was three- to sixfold more potent than that of WA-1 (WA-1 IC_50_ 70.9 ng/ml; variants IC_50_ 11.1 to 23.7 ng/ml) (fig. S3). Last, neutralization by A19-46.1 was similar to that of WA-1 for all variants except B.1.1.345 and B.1.177.31, which were still highly potent despite having IC_50_ values that were two to threefold less active (B.1.1.345, 95.0 ng/ml; B.1.177.311, 61.8 ng/ml; and WA-1, 39.8 ng/ml) (fig. S3). Together, these data show the capacity of these newly identified antibodies to maintain high neutralization potency against a diverse panel of 10 variant spike proteins.

## Antibody binding and neutralization of VOIs and VOCs

We analyzed neutralization of 13 circulating VOIs and VOCs, some of which have high transmissibility, including B.1.1.7, B.1.351, B.1.427, B.1.429, B.1.526, P.1, P.2, B.1.617.1, and B.1.617.2 (Fig. 2 and fig. S3) (*6*, *7*, *11*). Consistent with published data, we found that LY-CoV555, CB6, REGN10933, and REGN10987 maintained high potency against B.1.1.7 (IC_50_ 0.1 to 40.1 ng/ml), and LY-CoV555 and CB6 were unable to neutralize B.1.351 v1, B.1.351 v2, P.1 v1, or P.1 v2 variants (IC_50_ > 10,000 ng/ml) (Fig. 2 and fig. S3) (*12*, *14*, *26*); LY-CoV555 was unable to neutralize B.1.526 v2, B.1.617.1, and B.1.617.2; CB6 showed 5- to 27-fold worse activity against B.1.1.7+E484K and B.1.429+E484K but remained active against B.1.617.1 and B.1.617.2; REGN10933 showed 9- to 200-fold reduction in neutralization against variants with mutations at E484 (B.1.1.7+E484K, B.1.429+E484K, B.1.526 v2, P.1 v1/v2, and B.1.617.1) and maintained activity against B.1.617.2, which does not contain a mutation at E484 (Fig. 2 and fig. S3); and REGN10987 maintained or had slightly increased potency against each of the VOCs and VOIs except B.1.617.2, which showed a fourfold reduction in activity (Fig. 2 and fig. S3). In comparison, A23-58.1, B1-182.1, A19-46.1, and A19-61.1 maintained similar or improved potency (IC_50_ < 0.6 to 11.5 ng/ml) against B.1.1.7 and B.1.1.7+E484K relative to WA-1 (Fig. 2 and fig. S3). The potency of A19-46.1 was within 2.5-fold or lower relative to WA-1 for all variants (IC_50_ 11.5 to 101.4 ng/ml versus WA-1 39.8 ng/ml), except those containing L452R (IC_50_ >10,000 ng/ml) (B.1.427, B.1.429, B.1.429+E484K, B.1.617.1, and B.1.617.2) (Fig. 2 and fig. S3). Further analyses showed that A23-58.1, B1-182.1, and A19-61.1 maintained high potency against all VOCs and VOIs (IC_50_ < 0.6 to 28.3 ng/ml), including the recently identified B.1.617.1 and B.1.617.2 (Fig. 2 and fig. S3). These results indicate that despite being isolated from subjects infected with early ancestral SARS-CoV-2 viruses, each of these antibodies have highly potent reactivity against VOCs.

## Structural and functional analysis of VH1-58 antibodies

**Fig. 3.**
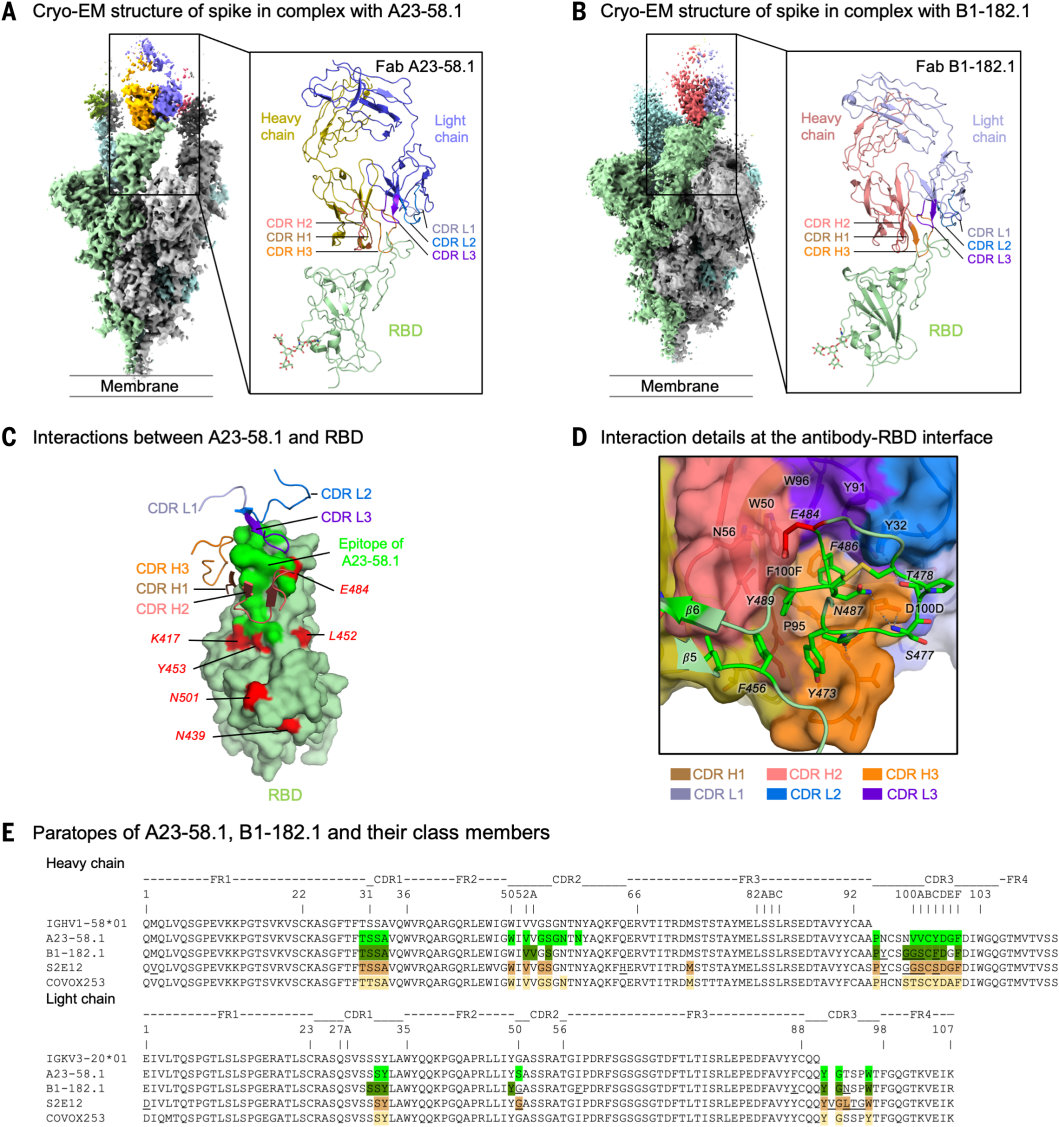
Structural basis of binding and neutralization for antibodies A23-58.1 and B1-182.1. (**A**) Cryo-EM structure of A23-58.1 Fab in complex with SARS-CoV-2 HexaPro spike. (Left) Overall density map. Protomers are light green, gray, and cyan. One of the A23-58.1 Fab bound to the RBD is shown in orange and blue. (Right) Structure of the RBD and A23-58.1 after local focused refinement. The heavy-chain CDRs are brown, salmon, and orange for CDR H1, CDR H2, and CDR H3, respectively. The light-chain CDRs are marine blue, light blue, and purple blue for CDR L1, CDR L2, and CDR L3, respectively. The contour level of the cryo-EM map is 5.7σ. (**B**) Cryo-EM structure of B1-182.1 Fab in complex with SARS-CoV-2 HexaPro spike. (Left) Overall density map. Protomers are light green, gray, and cyan. One of the B1-182.1 Fab bound to the RBD is shown in salmon and light blue. (Right) Structure of the RBD and B1-182.1 after local focused refinement. The heavy-chain CDRs are brown, deep salmon, and orange for CDR H1, CDR H2, and CDR H3, respectively. The light-chain CDRs are marine blue, slate, and purple blue for CDR L1, CDR L2, and CDR L3, respectively. The contour level of the cryo-EM map is 4.0σ. (**C**) Interaction between A23-58.1 and RBD. All CDRs were involved in binding of RBD. Epitope of A23-58.1 is shown in bright green surface. RBD mutations in current circulating SARS-CoV-2 variants are red. K417 and E484 are located at the edge of the epitope. (**D**) Interaction details at the antibody-RBD interface. The tip of the RBD binds to a crater formed by the CDRs (shown viewing down to the crater). Interactions between aromatic and hydrophobic residues are prominent at the lower part of the crater. Hydrogen bonds at the rim of the crater are indicated with dashed lines. RBD residues are indicated with italicized font. (**E**) Paratopes of A23-58.1, B1-182.1, S2E12 (PDB ID: 7K45), and COVOX253 (PDB ID: 7BEN) from the same germline. Sequences of B1-182.1, S2E12, and COVOX253 were aligned with variant residues underlined. Paratope residues for A23-58.1, B1-182.1, S2E12, and COVOX253 were highlighted in green, dark green, light brown, and light orange, respectively.

The two most potent antibodies, A23-58.1 and B1-182.1, shared highly similar gene family usage in their heavy and light chains, despite being from different donors (table S1). Both use IGHV1-58 heavy chains and IGKV3-20/IGKJ1 light chains and similarly low levels of somatic hypermutation (SHM) (<0.7%) (table S1). This antibody gene family combination has been identified in other COVID-19 convalescent subjects and has been proposed as a public clonotype (*27*–*30*). To gain structural insights on the interaction between this class of antibodies and the SARS-CoV-2 spike, we obtained cryo–electron microscopy (cryo-EM) reconstructions for structures of the Fab A23-58.1 bound to a stabilized WA-1 S at 3.39 Å resolution and of the Fab B1-182.1 bound to a stabilized WA-1 S at 3.15 Å resolution (Fig. 3, A and B; figs. S5 and S6; and table S2). This revealed that the antibody bound to spike with all RBDs in the up position, confirming the negative stain results (Fig. 1H). However, the cryo-EM reconstruction densities of the interface between RBD and Fab were poor owing to conformational variation.

To resolve the antibody-antigen interface, we performed local refinement and improved the local resolution to 3.89 Å for A23-58.1 and to 3.71 Å for B1-182.1 (figs. S5 and S6). Because both A23-58.1 and B1-182.1 recognized the RBD in a very similar way, we used the RBD-A23-58.1 structure for detailed analysis. Antibody A23-58.1 binds to an epitope on the RBD that faces the threefold axis of the spike and is accessible only in the RBD-up conformation (Fig. 3A). The interaction buried a total of 619 Å^2^ surface area from the antibody and 624 Å^2^ from the spike (table S3). The A23-58.1 paratope constituted all six complementarity-determining regions (CDRs) with heavy chain and light chain contributing 74 and 26% of the binding surface area, respectively (Fig. 3, C and E, and table S3). The 14-residue-long CDR H3, which is 48% of the heavy-chain paratope, kinks at Pro^95^ and Phe^100F^ (Kabat numbering scheme for antibody residues) to form a foot-like loop that is stabilized by an intraloop disulfide bond between Cys^97^ and Cys^100B^ at the arch. A glycan was observed at the CDR H3 Asn^96^ (fig. S5F). The CDRs formed an interfacial crater with a depth of ~10 Å and a diameter of ~20 Å at the opening. Paratope residues inside the crater were primarily aromatic or hydrophobic. CDR H3 Pro^95^ and Phe^100F^ lined the bottom, and CDR H1 Ala^33^, CDR H2 Trp^50^ and Val^52^, and CDR H3 Val^100A^ lined the heavy-chain side of the crater (Fig. 3, D and E). On the light-chain side, CDR L1 Tyr^32^ and CDR L3 residues Tyr^91^ and Trp^96^ provided 80% of the light chain–binding surface (Fig. 3, D and E). By contrast, paratope residues at the rim of the crater are mainly hydrophilic; for example, Asp^100D^ formed hydrogen bonds with Ser^477^ and Asn^487^ of the RBD (Fig. 3D and table S3).

**Fig. 4.**
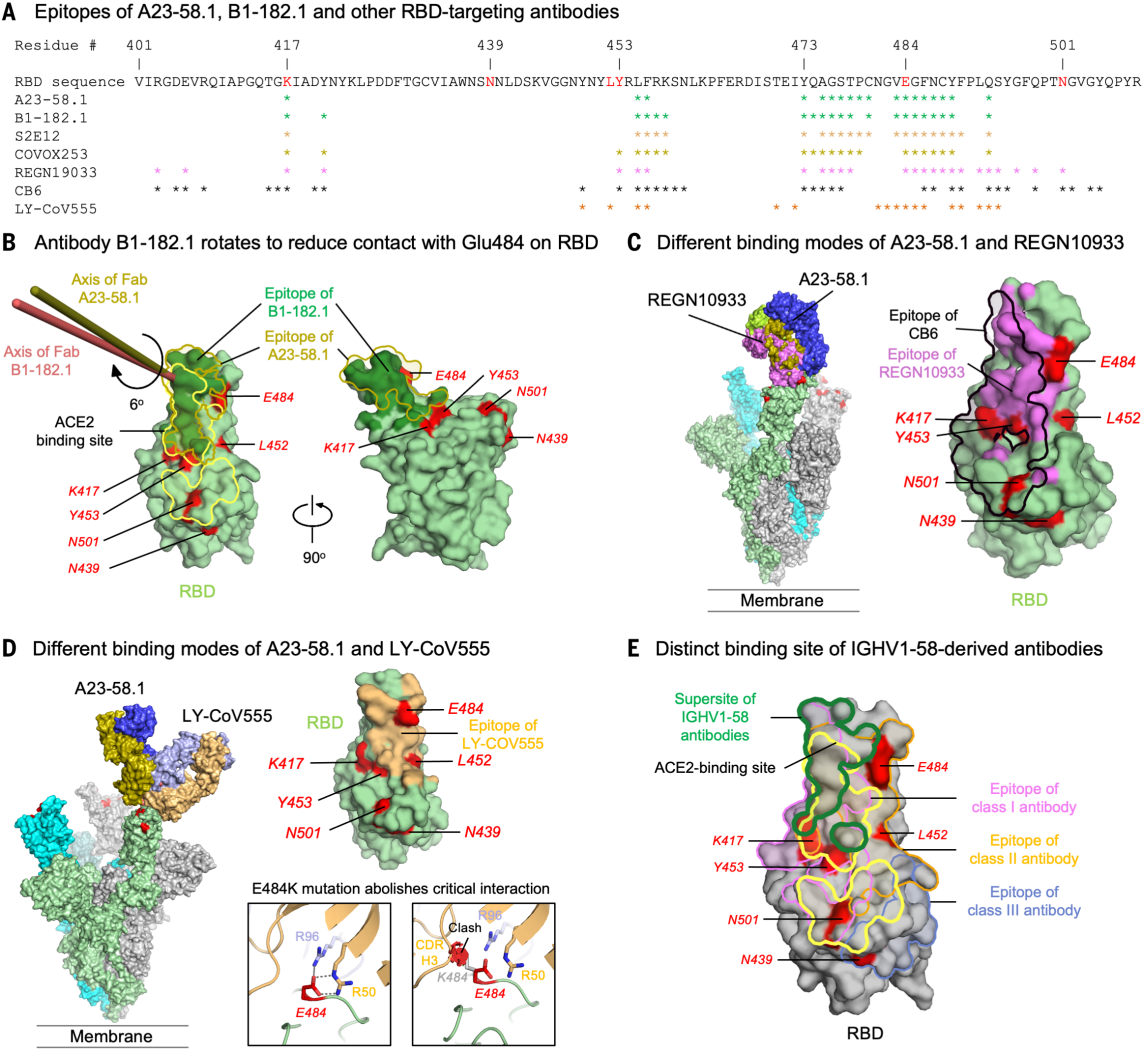
Binding modes of A23-58.1 and B1-182.1 enable neutralization to VOCs. (**A**) Mapping of epitopes of A23-58.1, B1-182.1, and other antibodies on RBD. Epitope residues for different RBD-targeting antibodies are marked with an asterisk under the RBD sequence. (**B**) Comparison of binding modes of A23-58.1 and B1-182.1. (Left) Analysis indicated that axis of Fab B1-182.1 is rotated 6° from that of A23-58.1. (Right) This rotation resulted in a slight shift of the epitope of B1-182.1 on RBD, which reduced its contact to E484. RBD mutations of concern are red, the epitope surface of B1-182.1 is dark green, and the borders of ACE2-binding site and A23-58.1 epitope are yellow and olive, respectively. (**C**) Comparison of binding modes of A23-58.1, CB6, and REGN10933. For clarity, one Fab is shown to bind to the RBD on the spike. The shift of the binding site to the saddle of RBD encircled K417, E484, and Y453 inside the CB6 (black line) and REGN10933 epitopes (violet surface), explaining their sensitivity to the K417N, Y453F, and E484K mutations. (**D**) Comparison of binding modes of A23-58.1 and LY-CoV555. (Left) One Fab is shown to bind to the RBD on the spike. (Top right) E484 is located inside the LY-CoV555 epitope. (Bottom right) E484K/Q mutation abolishes critical contacts between RBD and CDR H2 and CDR L3; moreover, E484K/Q and L452R cause potential clashes with heavy chain of LY-CoV555, explaining its sensitivity to the E484K/Q and L452R mutations. (**E**) IGHV1-58–derived antibodies target a supersite with minimal contacts to mutational hotspots. Supersite defined by common atoms contacted by the IGHV1-58–derived antibodies (A23-58.1, B1-182.1, S2E12, and COVOX253) on RBD is indicated with the green line. Boundaries of the ACE2-binding site and epitopes of class I, II, and III antibodies represented by C102 (PDB ID 7K8M), C144 (PDB ID 7K90), and C135 (PDB ID 7K8Z) are indicated with yellow, pink, light orange, and blue boundary lines, respectively.

The A23-58.1 epitope comprised residues between β5 and β6 at the tip of RBD (Figs. 3D and 4A). With the protruding Phe^486^ dipping into the crater formed by the CDRs, these residues formed a hook-like motif that is stabilized by an intraloop disulfide bond between Cys^480^ and Cys^488^. Aromatic residues—including Phe^456^, Tyr^473^, Phe^486^, and Tyr^489^—provided 48% (299 Å^2^) of the epitope (Fig. 3D and table S3). Lys^417^ and Glu^484^, which are located at the outer edge of the epitope, contributed only 3.7% of the binding surface (Fig. 3C and table S3). Overall, the cryo-EM analysis provides a structural basis for the potent neutralization of the E484K/Q mutant by A23-58.1.

The binding modes and sequences of A23-58.1 and B1-182.1 are very similar to those of previously reported IGHV1-58/IGKV3-20–derived antibodies, such as S2E12 (*27*), COVOX 253 (*30*), and CoV2-2196 (*31*), confirming that they are members of the same structural class (Fig. 3E). To understand why B1-182.1 is highly effective at neutralizing the emerging VOCs, we compared its binding mode with that of A23-58.1. Analysis indicated that B1-182.1 rotated about 6° along the long axis of Fab from that of A23-58.1 (Fig. 4B). This rotation on one hand increased B1-182.1 CDR L1 contacts on invariant regions of RBD to strengthen binding (Fig. 4B) and on the other hand critically reduced contact on Glu^484^ to 6 Å^2^ and main-chain only compared with ~40 Å^2^ main- and side-chain contacts for A58.1 and S2E12 (Fig. 4B and table S3). Overall, the subtle changes in antibody mode of recognition to regions on RBD harboring variant mutations provided structural basis on the effectiveness of B1-182.1 and A23-58.1 on neutralization of VOCs.

To understand how A23-58.1 and B1-182.1 overcome mutations that cause reduced antibody potency against virus variants, we superposed the antibody-RBD complex structures of CB6 [Protein Data Bank (PDB) ID 7C01] (*24*), REGN10933 (PDB ID 6XDG) (*25*, *26*), and LY-CoV555 (PDB ID 7KMG) (*19*) with the A23-58.1 structure over the RBD region. Both REGN10933 and CB6 bind to the same side of the RBD as does A23-58.1 (Fig. 4C). However, the binding surfaces of REGN10933 and CB6 were shifted toward the saddle of the open RBD and encompassed residues Lys^417^, Tyr^453^, Glu^484^, and Asn^501^ (Fig. 4C); mutations K417N and Y453F thus would abolish key interactions and lead to the loss of neutralization for both REGN10933 and CB6 (Fig. 2). By contrast, LY-CoV555 approached the RBD from a different angle, with its epitope encompassing Glu^484^ and Lys^452^ (Fig. 4D). Structural examination indicates that E484K/Q abolishes key interactions with CDR H2 Arg^50^ and CDR L3 Arg^96^ of LY-CoV555. In addition, both E484K/Q (Fig. 4D) and L452R mutations cause clashes with heavy chain of LY-CoV555. When compared with epitopes of class I, II, and III antibodies (*30*), the supersite defined by common contacts of the IGHV1-58–derived antibodies (A23-58.1, B1-182.1, S2E12, and COVOX253) had minimal interactions with residues at the mutational hotspots (Fig. 4E). These structural data suggest that the binding modes of A23-58.1 and B1-182.1 enabled their high effectiveness against the new SARS-CoV-2 VOCs.

**Fig. 5.**
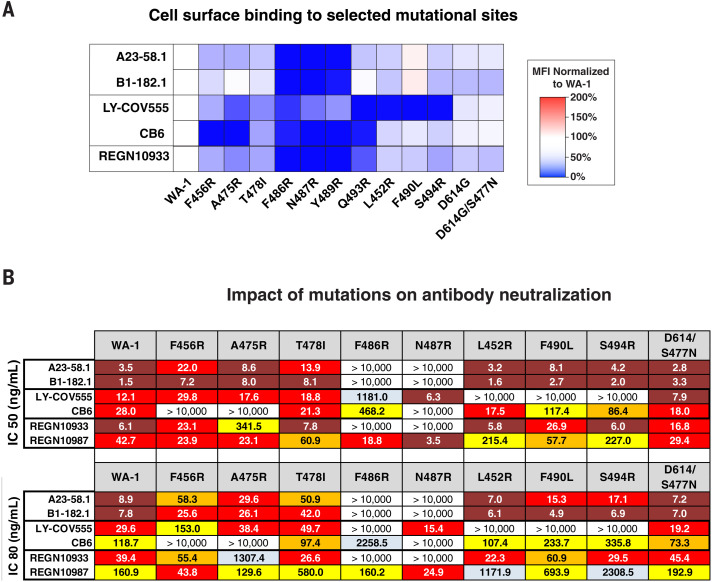
Critical binding residues for antibodies A23-58.1 and B1-182.1. (**A**) The indicated spike protein mutations predicted with structural analysis were expressed on the surface of HEK293 T cells, and binding to the indicated antibody was measured with flow cytometry. Data are shown as MFI normalized to the MFI for the same antibody against the WA-1 parental binding. Percent change is indicated by a color gradient from red (increased binding, max 200%) to white (no change, 100%) to blue (no binding, 0%). (**B**) IC_50_ and IC_80_ values for the indicated antibodies against WA-1 and the nine spike mutations. Ranges are indicated with white (>10,000 ng/ml), light blue (>1000 to ≤10,000 ng/ml), yellow (>100 to ≤1000 ng/ml), orange (>50 to ≤100 ng/ml), red (>10 to ≤50 ng/ml), and maroon (>1 to ≤10 ng/ml).

**Fig. 6.**
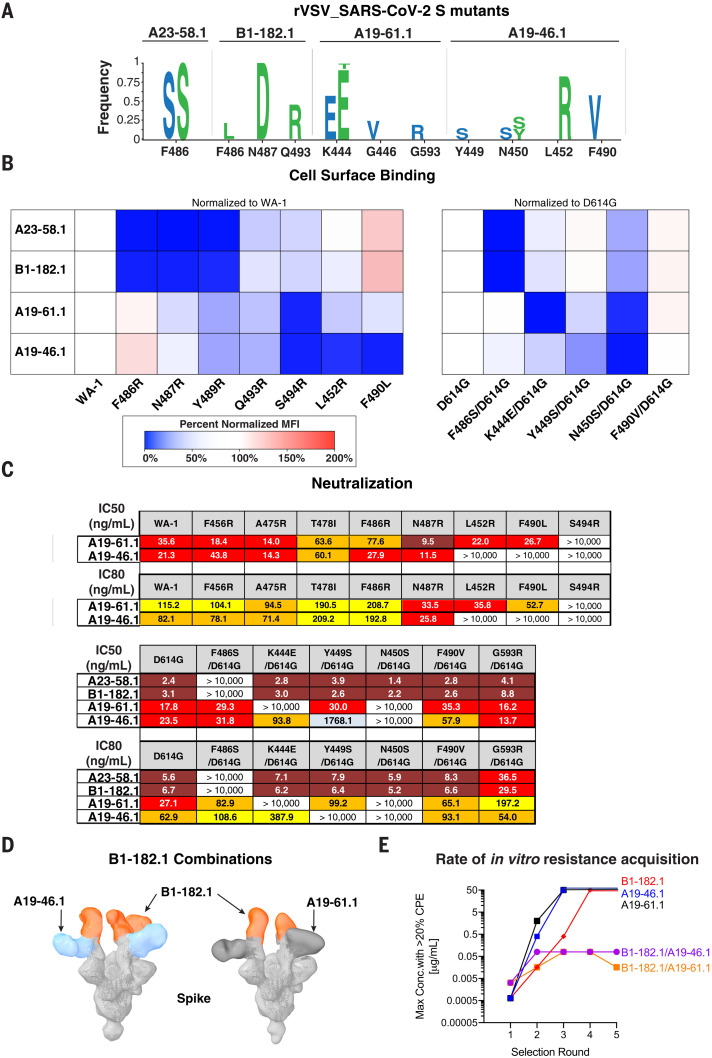
Mitigation of escape risk by using dual antibody combinations. (**A**) Replication competent vesicular stomatitis virus (rcVSV) whose genome-expressed SARS-CoV-2 WA-1 was incubated with serial dilutions of the indicated antibodies and wells with cytopathic effect (CPE) were passaged forward into subsequent rounds (fig. S8) after 48 to 72 hours. Total supernatant RNA was harvested, and viral genomes were shotgun sequenced to determine the frequency of amino acid changes. Shown are the spike protein amino acid and position change and frequency as a logo plot. Amino acid changes observed in two independent experiments are indicated in blue and green letters. (**B**) The indicated spike protein mutations predicted with structural analysis (Fig. 3) or observed with escape analysis (Fig. 6A) were expressed on the surface of HEK293 T cells, and binding to the indicated antibody was measured with flow cytometry. Data are shown as MFI normalized to the MFI for the same antibody against the (left) WA-1 or (right) D614G parental binding. Percent change is indicated with a color gradient from red (increased binding, max 200%) to white (no change, 100%) to blue (no binding, 0%). (**C**) IC_50_ and IC_80_ values for the indicated antibodies against WA-1 and the mutations predicted with structural analysis (Fig. 3) or observed with escape analysis (Fig. 6A). Ranges are indicated with white (>10,000 ng/ml), light blue (>1000 to ≤10,000 ng/ml), yellow (>100 to ≤1000 ng/ml), orange (>50 to ≤100 ng/ml), red (>10 to ≤50 ng/ml), and maroon (>1 to ≤10 ng/ml). (**D**) Negative-stain 3D reconstruction of the ternary complex of spike with Fab B1-182.1 and (left) A19-46.1 or (right) A19-61.1. (**E**) rcVSV SARS-CoV-2 was incubated with increasing concentrations (1.3 × 10^–4^ to 50 μg/ml) of either single antibodies (A19-46.1, A19-61.1, and B1-182.1) and combinations of antibodies (B1-182.1/A19-46.1 and B1-182.1/A19-61.1). Every 3 days, wells were assessed for CPE, and the highest concentration well with the >20% CPE was passaged forward onto fresh cells and antibody-containing media. Shown is the maximum concentration with >20% CPE for each of the test conditions in each round of selection. Once 50 μg/ml has been reached, virus was no longer passaged forward.

On the basis of the structural analysis, we investigated the relative contribution of predicted contact residues on binding and neutralization (Fig. 4A). Cell surface–expressed spike binding to A23-58.1 and B1-182.1 was knocked out by F486R, N487R, and Y489R (Fig. 5A and fig. S7), resulting in a lack of neutralization for viruses pseudotyped with spikes containing these mutations (Fig. 5B). By contrast, binding and neutralization of A19-46.1 and A19-61.1 were minimally affected by these changes (Fig. 6, B and C, and fig. S7). CB6, LY-CoV555, and REGN10933 binding and neutralization were also affected by the three mutations, which is consistent with the structural analysis that these residues are shared contact(s) with A23-58.1 and B1-182.1. Taken together, the shared binding and neutralization defects suggest that the hook-like motif and CDR crater are critical for the binding of antibodies within the VH1-58 public class.

## Generation and testing of escape mutations

To explore critical contact residues and mechanisms of escape that might be generated during the course of infection, we applied antibody selection pressure to replication-competent vesicular stomatitis virus (rcVSV) expressing the WA-1 SARS-CoV-2 spike (rcVSV-SARS2) (*32*) to identify spike mutations that confer in vitro resistance against A23-58.1, B1-182.1, A19-46.1, or A19-61.1 (fig. S8). rcVSV-SARS2 was incubated with increasing concentrations of antibody, and cultures from the highest concentration of antibody with >20% cytopathic effect (CPE), relative to no infection control, were carried forward into a second round of selection to drive resistance (fig. S8) (*26*). A shift to higher antibody concentrations required for neutralization indicates the presence of resistant viruses. To gain insight into spike mutations driving resistance, we performed Illumina-based shotgun sequencing (fig. S8). Variants present at a frequency of >5% and increasing from round 1 to round 2 were considered to be positively selected resistant viruses. For A19-46.1, escape mutations were generated at four sites: Y449S (frequency 15%), N450S (frequency 16%), N450Y (frequency 14%), L452R (frequency 83%), and F490V (frequency 58%) (Fig. 6A and fig. S8). The most dominant, L452R, is consistent with the previous finding that B.1.427, B.1.429, B.1.617.1, and B.1.617.2 were resistant to A19-46.1 (Fig. 2 and fig. S3). Although F490L severely reduced neutralization by A19-46.1 (IC_50_> 10,000 ng/ml), the effect of F490V was minimal, suggesting that F490V may require additional mutations for escape to occur (Fig. 6, A to C). Because Y449, N450, and L452 are immediately adjacent to S494, we tested whether S494R would also disrupt binding and neutralization (Fig. 6, A to C, and fig. S9) and found that this mutation mediates neutralization escape. Each of the identified residue locations was confirmed through binding and/or neutralization and would be expected to be accessible when RBD is in the up or down position (fig. S9), and several are shared by class II RBD antibodies (*18*, *33*) and REGN10933 (*25*, *34*).

Three residues were positively selected in the presence of A19-61.1: K444E/T (frequency 7-93%), G446V (frequency 24%), and G593R (frequency 19%) (Fig. 6A). There was no overlap with those selected by A19-46.1. G593R is located outside the RBD domain (fig. S9), did not affect neutralization, and may therefore represent a false positive. The highest frequency change was K444E, which represented 57 to 93% of the sequences in replicate experiments (Fig. 6A). This residue is critical for the binding of class III RBD antibodies such as REGN10987 (*18*, *25*, *26*, *34*). Because of the proximity of S494 to K444 and G446, S494R was tested for escape potential and shown to mediate escape from A19-61.1 neutralization. These results are consistent with A19-61.1 targeting a distinct epitope from REGN10987 and other class III RBD antibodies.

For A23-58.1, a single F486S mutation (frequency 91 to 98%) was positively selected. Similarly, B1-182.1 escape was mediated by F486L (frequency 21%), N487D (frequency 100%), and Q493R (frequency 45%). Q493R had minimal impact on binding and was not found to affect neutralization (Fig. 6, B and C). However, F486, N487, and Y489 were all in agreement with previous structural analysis (Figs. 3D, 5, and 6 and fig. S9). F486 is located at the tip of the RBD hook and contacts the binding interface in the antibody crater where aromatic side chains dominantly form the hook and crater interface (Fig. 3D). Therefore, the loss in activity may occur through replacement of a hydrophobic aromatic residue (phenylalanine) with a small polar side chain (serine) (Fig. 3D).

## Potential escape risk and mitigation

To probe the relevance of in vitro–derived resistance variants to potential clinical resistance, we investigated the relative frequency of variants containing escape mutations present in the GISAID sequence database using the COVID-19 Viral Genome Analysis Pipeline (https://cov.lanl.gov) (*22*) in which, as of 7 May 2021, there were 1,062,910 entries. Of the residues noted to mediate escape or resistance to A19-46.1 (Y449S, N450S/Y, L452R, F490L/V, and S494R), only F490L (0.02%) and L452R (2.27%) were present at greater than 0.01%. For the A19-61.1 escape mutations (K444E, G446V, and S494R), only G446V has been noted in the database >0.01% (0.03%). Last, for A23-58.1 and B1-182.1, ancestral WA-1 residues F486, N487, and Y489 were present in >99.96% of sequences, and only F486L was noted in the database at >0.01% (0.03%). Although the relative lack of A19-61.1, A23-58.1, and B1-182.1 escape mutations in circulating viruses could reflect either under-sampling or the absence of selection pressure, it may also suggest that the in vitro–derived mutations may exact a fitness cost on the virus.

Viral genome sequencing has suggested that in addition to spread through transmission, convergent selection of de novo mutations may be occurring (*6*–*9*, *13*, *22*, *35*). Therefore, effective therapeutic antibody approaches might require new antibodies or combinations of antibodies to mitigate the impact of mutations. On the basis of their complementary modes of spike recognition and breadth of neutralizing activity, combination of B1-182.1 with either A19-46.1 or A19-61.1 may decrease the rate of in vitro resistance acquisition compared with each antibody alone. Consistent with the competition data (Fig. 1F), negative-stain EM 3D reconstructions show that the Fabs in both combinations were able to simultaneously engage spike with the RBDs in the up position (Fig. 6D). Binding was observed for up to three Fabs of B1-182.1 and three Fabs of A19-46.1 or A19-61.1 per spike in the observed particles (Fig. 6D), indicating that the epitopes of A19-46.1 and A19-61.1 on the spike are accessible in both RBD up and down positions (Figs. 1H and 6D). The absence of observed RBD-down classes suggests the possibility that the combination induces a preferred mode of RBD-up engagement (RBD up versus RBD down) because of the requirement of B1-182.1 or A23-58.1 for RBD-up binding.

Next, we evaluated the capacity of individual antibodies or combinations to prevent the appearance of rcVSV SARS-CoV-2–induced cytopathic effect (CPE) through multiple rounds of passaging in the presence of increasing concentrations of antibodies. In each round, the well with the highest concentration of antibody with at least 20% CPE was carried forward into the next round. We found that wells with A19-61.1 or A785.46.1 single-antibody treatment reached the 20% CPE threshold in their 50 μg/ml well after three rounds of selection (Fig. 6E). Similarly, B1-182.1 single-antibody treatment reached >20% CPE in the 50 μg/ml wells after four rounds (Fig. 6E). Conversely, for both dual treatments (B1-182.1/A19-46.1 or B1-182.1/A19-61.1), the 20% CPE threshold was reached at a concentration of only 0.08 μg/ml and did not progress to higher concentrations, despite five rounds of passaging (Fig. 6E). Thus, combinations may lower the risk that a natural variant will lead to the complete loss of neutralizing activity and suggests a path forward for these antibodies as combination therapies.

## Discussion

Worldwide genomic sequencing has revealed the occurrence of SARS-CoV-2 variants that increase transmissibility and reduce potency of vaccine-induced and therapeutic antibodies (*10*–*16*). Recently, there has been substantial concern that antibody responses to natural infection and vaccination by using ancestral spike sequences may result in focused responses that lack potency against mutations present in more recent variants (such as K417N, L452R, T478K, E484K/Q, N501Y in B.1.351, B.1.617.1, and B.1.617.2) (*12*–*16*). Additionally, neutralization of P.1 viruses can be achieved by using sera obtained from subjects infected by B.1.351 (*36*), suggesting that shared epitopes in RBD (K417N, E484K, and N501Y) are mediating the cross-reactivity. Although the mechanism of B.1.351 and P.1 cross-reactivity is likely focused on the three RBD mutations, the mechanism of broadly neutralizing antibody responses between WA-1 and later variants is not as well established. As a first step to address the risk of reduced antibody potency against new variants, we isolated and defined new antibodies with neutralization breadth covering newly emerging SARS-CoV-2 variants, including the highly transmissible variants B.1.1.7, B.1.351, and B.1.617.2. Increased potency and breadth were mediated by binding to regions of the RBD tip that are offset from E484K/Q, L452R and other mutational hot spots that are major determinants of resistance in VOCs (*10*–*16*).

Our results show that highly potent neutralizing antibodies with activity against VOCs was present in at least three of four convalescent subjects who had been infected with ancestral variants of SARS-CoV-2 (Figs. 1 and 2 and figs. S3 and S10). Furthermore, our structural analyses, the relative paucity of potential escape variants in the GSAID genome database, the identification of public clonotypes (*27*, *28*), and each subject having mild to moderate illness all suggest that these antibodies were generated in subjects who rapidly controlled their infection and were not likely to have been generated because of the generation of a E484 escape mutation during the course of illness. Taken together, these data establish the rationale for a vaccine-boosting regimen that may be used to selectively induce immune responses that increase the breadth and potency of antibodies that target specific RBD regions of the spike glycoprotein (such as VH1-58 supersite). Because both variant sequence analysis and in vitro time-to-escape experiments suggest that combinations of these antibodies may have a lower risk for loss of neutralizing activity, these antibodies represent a potential means to achieve both breadth against current VOCs and to mitigate risk against those that may develop in the future.

## Materials and methods

### Isolation of PBMCs from SARS CoV-2 subjects

Human convalescent sera samples were obtained 25 to 55 days following symptom onset from adults with previous mild to moderate SARS-CoV-2 infection. Specimens were collected after subjects provided written informed consent under institutional review board approved protocols at the National Institutes of Health Clinical Center (NCT00067054) and University of Washington (Seattle) (Hospitalized or Ambulatory Adults with Respiratory Viral Infection [HAARVI] study). Whole blood was collected in vacutainer tubes, which were inverted gently to remix cells prior to standard Ficoll-Hypaque density gradient centrifugation (Pharmacia; Uppsala, Sweden) to isolate peripheral blood mononuclear cells (PBMCs). PBMCs were frozen in heat-inactivated fetal calf serum containing 10% dimethylsulfoxide in a Forma CryoMed cell freezer (Marietta, OH). Cells were stored at ≤–140°C

### Expression and purification of protein

For expression of soluble SARS CoV-2 S-2P protein, manufacturer’s instructions were followed. Briefly, plasmid was transfected using Expifectamine into Expi293 cells (Life Technology, #A14635, A14527) and the cultures enhanced 16-24 hours post-transfection. Following 4-5 days incubations at 120 rpm, 37°C, 9% CO2, supernatant was harvested, clarified via centrifugation, and buffer exchanged into 1X PBS. Protein of interests were then isolated by affinity chromatography using Streptactin resin (Life science) followed by size exclusion chromatography on a Superose 6 increase 10/300 column (GE healthcare).

Expression and purification of biotinylated S-2P, NTD, RBD-SD1 and Hexapro used in binding assays were produced by an in-column biotinylation method as previously described (*5*). Using full-length SARS-Cov2 S and human ACE2 cDNA ORF clone vector (Sino Biological, Inc) as the template to generate S1 or ACE2 dimer proteins. The S1 PCR fragment (1~681aa) was digested with Xbal and BamHI and cloned into the VRC8400 with HRV3C-his (6X) or Avi-HRV3C-his(6X) tag on the C-terminal. The ACE2 PCR fragment (1~740aa) was digested with Xbal and BamHI and cloned into the VRC8400 with Avi-HRV3C-single chain-human Fc-his (6x) tag on the C-terminal. All constructs were confirmed by sequencing. Proteins were expressed in Expi293 cells by transfection with expression vectors encoding corresponding genes. The transfected cells were cultured in shaker incubator at 120 rpm, 37°C, 9% CO2 for 4~5 days. Culture supernatants were harvested and filtered, and proteins were purified through a Hispur Ni-NTA resin (Thermo Scientific, #88221) and following a Hiload 16/600 Superdex 200 column (GE healthcare, Piscataway NJ) according to manufacturer’s instructions. The protein purity was confirmed with SDS–polyacrylamide gel electrophoresis (SDS-PAGE).

### Probe conjugation

SARS CoV-2 Spike trimer (S-2P) and subdomains (NTD, RBD-SD1, S1) were produced by transient transfection of 293 Freestyle cells as previously described (*4*). Avi-tagged S1 was biotinylated using the BirA biotin-protein ligase reaction kit (Avidity, #BirA500) according to the manufacturer’s instructions. The S-2P, RBD-SD1, and NTD proteins were produced by an in-column biotinylation method as previously described (*5*). Successful biotinylation was confirmed using Bio-Layer Interferometry, by testing the ability of biotinylated protein to bind to streptavidin sensors. Retention of antigenicity was confirmed by testing biotinylated proteins against a panel of cross-reactive SARS-CoV and SARS CoV-2 human monoclonal antibodies. Biotinylated probes were conjugated using either allophycocyanin (APC)-, Ax647-, BV421-, BV786-, BV711-, or BV570-labeled streptavidin. Reactions were prepared at a 4:1 molecular ratio of biotinylated protein to streptavidin, with every monomer labeled. Labeled streptavidin was added in ⅕ increments and in the dark at 4°C (rotating) for 20 min in between each addition. Optimal titers were determined using splenocytes from immunized mice and validated with SARS CoV-2 convalescent human PBMC.

### Isolation of and sequencing of antibodies by single B cell sorting

Cryopreserved human PBMCs from four COVID-19 convalescent donors were thawed and stained with Live/DEAD Fixable Aqua Dead Cell Stain kit (cat# L34957, ThermoFisher). After washing, cells were stained with a cocktail of anti-human antibodies, including CD3 (cat # 317332, Biolegend), CD8 (cat # 301048, Biolegend), CD56 (cat # 318340, Biolegend), CD14 (cat # 301842, Biolegend), CD19 (cat # IM2708U, Beckman Coulter), CD20 (cat # 302314, Biolegend), IgG (cat # 555786, BD Biosciences), IgA (cat # 130-114-001, Miltenyi), IgM (cat # 561285, BD Biosciences) and subsequently stained with fluorescently labeled SARS-CoV-2 S-2P (APC or Ax647), S1 (BV786 or BV570), RBD-SD1 (BV421) and NTD (BV711 or BV421) probes. Antigen-specific memory B cells (CD3-CD19^+^CD20^+^IgG^+^ or IgA^+^ and S-2P^+^ and/or RBD+ for the donors Subjects A19, A20 and A23, S-2P^+^ and/or NTD^+^ for the donor Subject B1) were sorted using a FACSymphony S6 (BD Sciences) into Buffer TCL (Qiagen) with 1% 2-mercaptoethanol (ThermoFisher Scientific). Nucleic acids were purified using RNAClean magnetic beads (Beckman Coulter) followed by reverse transcription using oligo-dT linked to a custom adapter sequence and template switching using SMARTScribe RT (Takara). PCR amplification was carried out using SeqAmp DNA Polymerase (Takara). A portion of the amplified cDNA was enriched for B cell receptor sequences using forward primers complementary to the template switch oligo and reverse primers against the IgA (GAGGCTCAGCGGGAAGACCTTGGGGCTGGTCGG) IgG, Igκ, and Igλ (*37*) constant regions. Enriched products were made into Illumina-ready sequencing libraries using the Nextera XT DNA Library Kit with Unique Dual Indexes (Illumina). The Illumina-ready libraries were sequenced by paired end 150 cycle MiSeq reads. The resulting reads were demultiplexed using an in-house script and V(D)J sequences were assembled using BALDR in unfiltered mode (*38*). Poor or incomplete assemblies or those with low read support were removed, and the filtered contigs were re-annotated with SONAR v4.2 in single cell mode (*39*). A subset of the final antibodies was manually selected for synthesis based on multiple considerations, including gene usage, SHM levels, CDRH3 length, convergent rearrangements, and specificity implied by flow cytometry.

### Synthesis, cloning, and expression of monoclonal antibodies

Sequences were selected for synthesis to sample expanded clonal lineages within our dataset and convergent rearrangements both among donors in our cohort and compared to the public literature. In addition, we synthesized a variety of sequences designed to be representative of the whole dataset along several dimensions, including apparent epitope based on flow data; V gene usage; SHM levels; CDRH3 length; and isotype. Variable heavy chain sequences were human codon optimized, synthesized and cloned into a VRC8400 (CMV/R expression vector)-based IgG1 vector containing an HRV3C protease site (*40*) as previously described (*36*). Similarly, variable lambda and kappa light chain sequences were human codon optimized, synthesized and cloned into CMV/R-based lambda or kappa chain expression vectors, as appropriate (Genscript). Previously published antibody vectors for LY-COV555(18) and mAb114 (*41*) were used. The antibodies: REGN10933 was produced from published sequences (*25*) and kindly provided by Devin Sok from Scripps. For antibodies where vectors were unavailable (e.g., S309, CB6), published amino acids sequences were used for synthesis and cloning into corresponding pVRC8400 vectors (*42*, *43*). For antibody expression, equal amounts of heavy and light chain plasmid DNA were transfected into Expi293 cells (Life Technology) by using Expi293 transfection reagent (Life Technology). The transfected cells were cultured in shaker incubator at 120 rpm, 37°C, 9% CO2 for 4~5 days. Culture supernatants were harvested and filtered, mAbs were purified over Protein A (GE Health Science) columns. Each antibody was eluted with IgG elution buffer (Pierce) and immediately neutralized with one tenth volume of 1M Tris-HCL pH 8.0. The antibodies were then buffer exchanged as least twice in PBS by dialysis.

### ELISA method description

Testing is performed using the automated enzyme-linked immunosorbent assay (ELISA) method as detailed in VRC-VIP SOP 5500 Automated ELISA on Integrated Automation System. Quantification of IgG concentrations in serum/plasma are performed with a Beckman Biomek based automation platform. The SARS-CoV-2 S-2P (VRC-SARS-CoV-2 S-2P (15-1208)-3C-His8-Strep2x2) and RBD (Ragon-SARS-CoV-2 S-RBD (319-529)-His8-SBP) Antigen are coated onto Immulon 4HBX flat bottom plates overnight for 16 hours at 4°C at a concentration of 2 μg/ml and 4μg/ml, respectively. Proteins were produced and generously provided by Dr. Dominic Esposito (Frederick National Laboratory for Cancer Research, NCI). Antigen concentrations were defined during assay development and antigen lot titration. Plates are washed and blocked (3% milk TPBS) for 1 hour at room temperature. Duplicate serial 4-fold dilutions covering the range of 1:100 to 1:1638400 (8-dilution series) of the test sample (diluted in 1%milk in TPBS) are incubated at room temperature for 2 hours followed by Horseradish Peroxidase - labeled goat anti-human antibody detection (1 hour at room temperature) (Thermo Fisher catalogue # A1881), and TMB substrate (15 min at room temperature; DAKO catalogue # S1599) addition. Color development is stopped by addition of sulfuric acid and plates are read within 30 min at 450 nm and 650 nm via the Molecular Devices Paradigm plate reader. Each plate harbors a negative control (assay diluent), positive control (SARS-CoV-2 S2-specific monoclonal antibody S-652-112 spiked in NHS and/or pool of COVID-19 convalescent sera) and batches of 5 specimen run in duplicates. All controls are trended over time.

Endpoint Titer dilution from raw OD data are interpolated using the plate background OD + 10 STDEV by asymmetric sigmoidal 5-pl curve fit of the test sample. In the rare event, the asymmetric sigmoidal 5-pl curve failed to interpolate the endpoint titer, a sigmoidal 4-pl curve is used for the analysis. Area under the curve (AUC) is calculated with baseline anchored by the plate background OD + 10 STDEV. Data analysis is performed using Microsoft Excel and GraphPad Prism Version 8.0.

### Assignment of major binding determinant using MSD binding assay

MSD 384-well streptavidin-coated plates (MSD, cat# L21SA) were blocked with MSD 5% Blocker A solution (MSD, cat# R93AA), using 35 ul per well. These plates were then incubated for 30 to 60 min at room temperature. Plates were washed with 1x Phosphate Buffered Saline + 0.05% Tween 20 (PBST) on a Biotek 405TS automated microplate washer. Five SARS CoV-2 capture antigens were used. Capture antigens consisted of VRC-produced S1, S-2P, S6P (Hexapro), RBD, and NTD. All antigens were AVI-tag biotinylated using BirA (Avidity, cat # BirA500) AVI-tag specific biotinylation following manufacturer’s instructions except S1. For S1, an Invitrogen FluoReporter Mini-Biotin-XX Protein Labeling Kit (Thermo Fisher, cat # F6347) was utilized to achieve random biotinylation. Antigen coating solutions were prepared for S1, S-2P, S6P, RBD, and NTD at optimized concentrations of 0.5, 0.25, 1, 0.5, and 0.25 ug/ml, respectively. These solutions were then added to MSD 384-well plates, using 10 μl per well. Each full antigen set is intended to test one plate of experimental SARS CoV-2 monoclonal antibodies (mAbs) at one dilution. Once capture antigen solutions were added, plates were incubated for 1 hour at room temperature on a Heidolph Titramax 1000 (Heidolph, part # 544-12200-00) vibrational plate shaker at 1000 rpm. During this time, experimental SARS CoV-2 mAb dilution plates were prepared. Using this initial plate, 3 dilution plates were created at dilution factors of 1:100, 1:1000, and 1:10000. Dilutions were performed in 1% assay diluent (MSD 5% Blocker A solution diluted 1:5 in PBST). Positive control mAbs S652-109 (SARS Cov-2 RBD specific) and S652-112 (SARS CoV-2 S1, S-2P, S6P, and NTD specific) and negative control mAb VRC01 (anti-HIV) were added to all dilution plates at a uniform concentration of 0.05 μg/ml. Once mAb dilution plates were prepared, MSD 384-well plates were washed as above. The content of each 96-well dilution plate was added to the MSD 384-well plates, using 10 μl per well. MSD 384-well plates were then incubated for 1 hour at room temperature on vibrational plate shaker at 1000 rpm. MSD 384-well plates were washed as above, and MSD Sulfo-Tag labeled goat anti-human secondary detection antibody (MSD, cat# R32AJ) solution was added to plates at a concentration of 0.5 ug/ml, using 10 μl per well. Plates were again incubated for 1 hour at room temperature on vibrational plate shaker at 1000 rpm. MSD 1x Read Buffer T (MSD, cat# R92TC) was added to MSD 384-well plates, using 35 μl per well. MSD 384-well plates were then read using MSD Sector S 600 imager. Gross binding epitope of S-2P or Hexapro positive antibodies was assigned into the following groups: RBD (i.e., RBD^+^ or RBD^+^/S1^+^ AND NTD^–^), NTD (i.e., NTD^+^ or NTD^+^/S1^+^ and RBD^–^), S2 (i.e., S1^–^, RBD^–^ and NTD^–^) or indeterminant (i.e., mixed positive). Antibodies lacking binding to any of the antigens were assigned to the “no binding” group.

### Full-length S constructs

cDNAs encoding full-length S from SARS CoV-2 (GenBank ID: QHD43416.1) were synthesized, cloned into the mammalian expression vector VRC8400 (*42*, *43*) and confirmed by sequencing. S containing D614G amino acid change was generated using the wt S sequence. Other variants containing single or multiple aa changes in the S gene from the S wt or D614G were made by mutagenesis using QuickChange lightning Multi Site-Directed Mutagenesis Kit (cat # 210515, Agilent). The S variants, N439K, Y453F, A222V, E484K, K417N, S477N, N501Y, delH69/V70, N501Y-delH69/V70, N501Y-E484K-K417N, B.1.1.7 (H69del-V70del-Y144del-N501Y-A570D-P681H-T716I-S982A-D1118H), B.1.351.v1 (L18F-D80A-D215G-(L242-244)del-R246I-K417N-E484K-N501Y-A701V), B.1.351.v2 (L18F-D80A-D215G-(L242-244)del-K417N-E484K-N501Y-A701V), B.1.427 (L452R-D614G), B.1.429 (S13I-W152C-L452R-D614G), B.1.526.v2 (L5F-T95I-D253G-E484K-D614G-A701V), P.1.v1 (L18F-T20N-P26S-D138Y-R190S-K417T-E484K-N501Y-D614G-H655Y-T1027I), P.1.v2 (L18F-T20N-P26S-D138Y-R190S-K417T-E484K-N501Y-D614G-H655Y-T1027I-V7116F), P.2 (E484K-D614G-V7116F), B.1.617.1 (T95I-G412D-E154K-L452R-E484Q-D614G-P681R-Q1071H), B.1.617.2 (T19R-G142D-del156-157-R158G-L452R-T478K-D614G-P681R-D950N) and antibody escape mutations, F486S, K444E, Y449S, N450S and F490V were generated based on S D614G while the antibody contact residue mutations, F456R, A475R, T478I, F486R, Y489R, N487R, L452R, F490L, Q493R, S494R on S wt. These full-length S plasmids were used for pseudovirus production and for cell surface binding assays.

### Pseudovirus neutralization assay

S-containing lentiviral pseudovirions were produced by co-transfection of packaging plasmid pCMVdR8.2, transducing plasmid pHR’ CMV-Luc, a TMPRSS2 plasmid and S plasmids from SARS CoV-2 variants into 293T (ATCC) cells using Fugene 6 transfection reagent (Promega, Madison, WI) (*44*–*46*). 293T-ACE2 cells, provided by Dr. Michael Farzan, were plated into 96-well white/black Isoplates (PerkinElmer, Waltham, MA) at 5000 cells per well the day before infection of SARS CoV-2 pseudovirus. Serial dilutions of mAbs were mixed with titrated pseudovirus, incubated for 45 min at 37°C and added to 293T-ACE2 cells in triplicate. Following 2 hours of incubation, wells were replenished with 150 ml of fresh media. Cells were lysed 72 hours later, and luciferase activity was measured with Microbeta (Perking Elmer). Percent neutralization and neutralization IC50s, IC80s were calculated using GraphPad Prism 8.0.2. Serum neutralization assays were performed as above excepting all human sera had an input starting serial dilution of 1:20 and neutralization was quantified as the inhibition dilution 50% (ID_50_) of virus entry. Alternative method pseudovirus neutralization assay in fig. S3 utilized a first-generation lentivirus system and was performed as in Wibmer *et al*. (*12*).

### Cell surface binding

Human embryonic kidney (HEK) 293 T cells were transiently transfected with plasmids encoding full length SARS CoV-2 spike variants using lipofectamine 3000 (L3000-001, ThermoFisher) following manufacturer’s protocol. After 40 hours, the cells were harvested and incubated with monoclonal antibodies (1 μg/ml) for 30 min. After incubation with the antibodies, the cells were washed and incubated with an allophycocyanin conjugated anti-human IgG (709-136-149, Jackson Immunoresearch Laboratories) for another 30 min. The cells were then washed and fixed with 1% paraformaldehyde (15712-S, Electron Microscopy Sciences). The samples were then acquired in a BD LSRFortessa X-50 flow cytometer (BD biosciences) and analyzed using Flowjo (BD biosciences). Mean fluorescent intensity (MFI) for antibody binding to S wt or D614G was set up as 100%. The MFI of the antibody binding to each variant was normalized to S wt or D614G.

### Competitive mAb binding assay using surface plasmon resonance

Monoclonal antibody (mAb) competition assays were performed on a Biacore 8K+ (Cytiva) surface plasmon resonance spectrometer. Anti-histidine IgG_1_ antibody was immobilized on Series S Sensor Chip CM5 (Cytiva) using a His capture kit (Cytiva), per manufacturer’s instructions. 1X PBS-P+ (Cytiva) was used for running buffer and diluent, unless noted. 8X His-tagged SARS-CoV-2 Spike protein containing 2 proline stabilization mutations, K986P and V987P, (S-2P) (*4*) was captured on the active sensor surface. “Competitor” mAb or a negative control mAb114 (*37*) were first injected over both active and reference surfaces, followed by “analyte” mAb. Between cycles, sensor surfaces were regenerated with 10 mM glycine, pH 1.5 (Cytiva).

For data analysis, sensorgrams were aligned to Y (Response Units, RUs) = 0, beginning at the beginning of each mAb binding phase in Biacore 8K Insights Evaluation Software (Cytiva). Reference-subtracted, relative “analyte binding late” report points (in RUs) were used to determine percent competition for each mAb. Maximum analyte binding for each mAb was first defined by change in RUs during analyte binding phase when negative control mAb was used as competitor mAb. Percent competition (%C) was calculated using the following formula: %C = 100 * {1 –[((analyte mAb binding RUs when S-2P-specific mAb is used as competitor) / (maximum analyte binding RUs when negative control mAb is used as competitor)]}.

### Competitive ACE2 binding assay using biolayer interferometry

Antibody cross-competition was determined based on biolayer interferometry using a fortéBio Octet HTX instrument. His1K biosensors (fortéBio) were equilibrated for >600 s in Blocking Buffer [1% BSA (Sigma) + 0.01% Tween-20 (Sigma) + 0.01% Sodium Azide (Sigma) + PBS (Gibco), pH7.4] prior to loading with his tagged S-2P protein (10 μg/ml in Blocking Buffer) for 1200s. Following loading, sensors were incubated for 420s in Blocking Buffer prior to incubation with competitor mAbs (30 mg/ml in Blocking Buffer) or ACE2 (266 nM in Blocking Buffer) for 1200s. Sensors were then incubated in Blocking buffer for 30s prior to incubation with ACE2 (266 nM in Blocking Buffer) for 1200s. Percent competition (PC) of ACE2 mAbs binding to competitor-bound S-2P was determined using the equation: PC = 100 − [(ACE2 binding in the presence competitor mAb) ⁄ (ACE2 binding in the absence of competitor mAb)] × 100. All the assays were performed in duplicate and with agitation set to 1000 rpm at 30°C.

### Inhibition of S protein binding to cell surface ACE2

Serial dilutions of mAb were mixed with pre-titrated biotinylated S trimer (S-2P), incubated for 30 min at RT and added to BHK21 cells stably expressing hACE2 on cell surface. Following 30 min of incubation on ice, the cells were washed and incubated with an BV421 conjugated Streptavidin (cat # 563259, BD Biosciences) for another 30 min. The cells were then washed and fixed with 1% paraformaldehyde (15712-S, Electron Microscopy Sciences). The samples were then acquired in a BD LSRFortessa X-50 flow cytometer (BD biosciences) and analyzed using Flowjo (BD biosciences). MFI for S protein binding to cell surface was set up as 100%. Percent inhibition of S protein binding to cell surface ACE2 by mAb IgG and half-maximal effective concentration (EC_50_) were calculated using GraphPad Prism 8.0.2.

### Live virus neutralization assay

Full-length SARS CoV-2 virus based on the Seattle Washington strain was designed to express nanoluciferase (nLuc) and was recovered via reverse genetics and described previously (17). Virus titers were measured in Vero E6 USAMRIID cells, as defined by plaque forming units (PFU) per ml, in a 6-well plate format in quadruplicate biological replicates for accuracy. For the 96-well neutralization assay, Vero E6 USAMRID cells were plated at 20,000 cells per well the day prior in clear bottom black walled plates. Cells were inspected to ensure confluency on the day of assay. Serially diluted mAbs were mixed in equal volume with diluted virus. Antibody-virus and virus only mixtures were then incubated at 37°C with 5% CO_2_ for one hour. Following incubation, serially diluted mAbs and virus only controls were added in duplicate to the cells at 75 PFU at 37°C with 5% CO_2_. After 24 hours, cells were lysed, and luciferase activity was measured via Nano-Glo Luciferase Assay System (Promega) according to the manufacturer specifications. Luminescence was measured by a Spectramax M3 plate reader (Molecular Devices, San Jose, CA). Virus neutralization titers were defined as the sample dilution at which a 50% reduction in RLU was observed relative to the average of the virus control wells.

Live virus neutralization assays described above were performed with approved standard operating procedures for SARS CoV-2 in a biosafety level 3 (BSL-3) facility conforming to requirements recommended in the Microbiological and Biomedical Laboratories, by the US Department of Health and Human Service, the US Public Health Service, and the US Center for Disease Control and Prevention (CDC), and the National Institutes of Health (NIH).

### Production of Fab fragments from monoclonal antibodies

To generate mAb-Fab, IgG was incubated with HRV3C protease (EMD Millipore) at a ratio of 100 units per 10 mg IgG with HRV 3C Protease Cleavage Buffer (150 mM NaCl, 50 mM Tris-HCl, pH 7.5) at 4°C overnight. Fab was purified by collecting flowthrough from Protein A column (GE Health Science), and Fab purity was confirmed by SDS-PAGE.

### Determination of binding kinetics of Fab

A fortéBio Octet HTX instrument was used to measure binding kinetics of the Fab of A23-58.1, B1-182.1, A19-46.1 and A19-61.1 to SARS CoV-2 S-2P protein. SA biosensors (fortéBio) were equilibrated for >600 s in Blocking Buffer [1% BSA (Sigma) + 0.01% Tween-20 (Sigma) + 0.01% Sodium Azide (Sigma) + PBS (Gibco), pH7.4] prior to loading with biotinylated S-2P protein (1.5 mg/ml in Blocking Buffer) for 600s. Following loading, sensors were incubated for 420s in Blocking Buffer prior to binding assessment of the Fabs. Association of Fabs was measured for 300 s and dissociation was measured for up to 3600 s in Blocking Buffer. All the assays were performed with agitation set to 1000 rpm at 30°C. Data analysis and curve fitting were carried out using Octet analysis software, version 11-12. Experimental data were fitted using a 1:1 binding model. Global analyses of the complete data sets assuming binding was reversible (full dissociation) were carried out using nonlinear least-squares fitting allowing a single set of binding parameters to be obtained simultaneously for all concentrations used in each experiment.

### Negative-stain electron microscopy.

Protein samples were diluted to a concentration of approximately 0.02 mg/ml with 10 mM HEPES, pH 7.4, supplemented with 150 mM NaCl. A 4.8-μl drop of the diluted sample was placed on a freshly glow-discharged carbon-coated copper grid for 15 s. The drop was then removed with filter paper, and the grid was washed with three drops of the same buffer. Protein molecules adsorbed to the carbon were negatively stained by applying consecutively three drops of 0.75% uranyl formate, and the grid was allowed to air-dry. Datasets were collected using a Thermo Scientific Talos F200C transmission electron microscope operated at 200 kV and equipped with a Ceta camera. The nominal magnification was 57,000x, corresponding to a pixel size of 2.53 Å, and the defocus was set at -1.2 μm. Data was collected automatically using EPU. Single particle analysis was performed using CryoSPARC (47).

### Cryo-EM specimen preparation and data collection.

The stabilized SARS CoV-2 spike HexaPro (3) was mixed with Fab A23-58.1 or B1-182.1 at a molar ratio of 1.2 Fab per protomer in PBS. The final spike protein concentration was 0.5 mg/ml. n-Dodecyl β-D-maltoside (DDM) detergent was added shortly before vitrification to a concentration of 0.005%. Quantifoil R 2/2 gold grids were subjected to glow discharging in a PELCO easiGlow device (air pressure, 0.39 mBar; current, 20 mA; duration, 30 s) immediately before specimen preparation. Cryo-EM grids were prepared using an FEI Vitrobot Mark IV plunger with the following settings: chamber temperature of 4°C, chamber humidity of 95%, blotting force of –5, blotting time of 3 s, and drop volume of 2.7 μl. Datasets were collected at the National CryoEM Facility (NCEF), National Cancer Institute, on a Thermo Scientific Titan Krios G3 electron microscope equipped with a Gatan Quantum GIF energy filter (slit width: 20 eV) and a Gatan K3 direct electron detector (table S2). Four movies per hole were recorded in the counting mode using Latitude software. The dose rate was 14.65 *e*^–^/s/pixel.

### Cryo-EM data processing and model fitting

Data process workflow, including Motion correction, CTF estimation, particle picking and extraction, 2D classification, ab initio reconstruction, homogeneous refinement, heterogeneous refinement, non-uniform refinement, local refinement and local resolution estimation, were carried out with C1 symmetry in cryoSPARC 2.15 (*47*) For local refinement to resolve the RBD-antibody interface, a mask for the entire spike-antibody complex without the RBD-antibody region was used to extract the particles and a mask encompassing the RBD-antibody region was used for refinement. The overall resolution was 3.39 Å and 3.15 Å for the map of A23-58.1- and B1-182.1-bound spike, 3.89 Å and 3.71 Å for the map of RBD:antibody interface after local refinement, respectively. The coordinates for the SARS-CoV-2 spike with three ACE2 molecules bound at pH 7.4 (PDB ID: 7KMS) were used as initial models for fitting the cryo-EM map. Iterative manual model building and real space refinement were carried out in Coot (*48*) and in Phenix (*49*), respectively. Molprobity (*50*) was used to validate geometry and check structure quality at each iteration step. UCSF Chimera and ChimeraX were used for map fitting and manipulation (*51*).

### Selection of rcVSV SARS CoV-2 virus escape variants using monoclonal antibodies

A replication competent vesicular stomatitis virus (rcVSV) with its native glycoprotein replaced by the Wuhan-1 spike protein (rcVSV SARS CoV-2) that contains a 21 amino acid deletion at the C-terminal region (*32*) (generous gift of Kartik Chandran and Rohit Jangra). Passage 7 virus was passaged twice on Vero cells to obtain a polyclonal stock. A single plaque from this 9th passage was double plaque purified and expanded on Vero cells to create monoclonal virus population. The reference genome for this stock was sequence using Illumina-based sequencing as described below.

To select for virus escape variants, an equal volume of clonal population of rcVSV SARS CoV-2 was mixed with serial dilutions of antibodies (5-fold) in DMEM supplemented with 10% FCS and Glutamine to give an MOI of 0.1 to 0.001 at the desired final antibody concentration (range 5.1e^–6^ to 50 μg/ml and 0 μg/ml). Virus:antibody mixtures were incubated at room temperature for 1 hour. After incubation, 300 μl of virus:antibody mixtures were added to 1 × 10^5^ Vero E6 cells in 12 well plates for 1 hour at 37°C, 5% CO_2_. The plates were rotated every 15 min to prevent drying. After absorption, 700 μl of additional antibodies mixture was added to each well at their respective concentration. Cells were incubated for 72hrs at 37°C, 5% CO_2_. Virus replication was monitored using cytopathic effect and supernatant was collected from the wells with cytopathic effect. Harvested supernatant was clarified by centrifugation at 3750rpm for 10 min. For the subsequent rounds of selection, clarified supernatant from the well with the highest concentration of antibody that has CPE >20% supernatant was diluted prior to being mixed with equal volume of antibodies as in the initial round of selection. Infection, monitoring and collection of supernatants was performed as in the initial round.

### Shotgun sequencing of rcVSV SARS CoV2 supernatants

Total RNA was extracted from clarified supernatants using QIAmp viral RNA mini extraction kit (Qiagen) following the manufacturer’s recommended protocol. Purified RNA was fragmented using NEBNext Ultra II RNA Library Prep reagents, then reverse transcribed using random hexamers, and double-stranded cDNA was synthesized (New England BioLabs) as previously described (*52*). Double-stranded cDNA was purified using magnetic beads (MagBio Genomics) and barcoded Illumina-ready libraries were subsequently prepared (New England BioLabs). The libraries were sequenced as paired-end 2x150 base pair NextSeq 2000 reads.

### Spike SNP variant calls of rcVSV antibody induced revertants

Raw sequencing reads were demultiplexed and trimmed to remove adaptor sequences and low quality bases. They were then aligned against the reference viral genome with Bowtie (v2.4.2). Single nucleotide polymorphisms (SNPs) were called using HaplotypeCaller from the Genome Analysis Tool Kit (GATK, v4.1.9.0). The HaplotypeCaller parameter, “–sample-ploidy”, was set to 100 in order to identify SNPs with a prevalence of at least 1%. SNPs for all samples were then aggregated, interrogated and translated using custom scripts. A SNP and correlated amino acid translation for the spike protein was considered positive if it was present at a frequency of greater than 0.1 (10%) and showed an increasing frequency from round 1 to round 2 of the antibody selections.

### Multiplex SARS-CoV-2 variant binding assay

Multiplexed Plates (96 well) precoated with SARS Cov2 spike (WA-1), SARS Cov2 RBD (WA-1), SARS Cov2 spike (B.1.351), SARS Cov2 spike (B.1.1.7), SARS Cov2 spike (P.1), SARS Cov2 RBD (B.1.351), SARS Cov2 RBD (B.1.1.7), SARS Cov2 RBD (P.1) and BSA are supplied by the manufacturer. On the day of the assay, the plate is blocked for 60 min with MSD Blocker A (5% BSA). The blocking solution is washed off and test samples are applied to the wells at 4 dilution (1:100, 1:500, 1:2500, and 1:10,000) unless otherwise specified and allowed to incubate with shaking for two hours. Plates are washed and Sulfo-tag labeled anti IgG antibody is applied to the wells and allowed to associate with complexed coated antigen – sample antibody within the assay wells. Plates are washed to remove unbound detection antibody. A read solution containing ECL substrate is applied to the wells, and the plate is entered into the MSD Sector instrument. A current is applied to the plate and areas of well surface where sample antibody has complexed with coated antigen and labeled reporter will emit light in the presence of the ECL substrate. The MSD Sector instrument quantitates the amount of light emitted and reports this ECL unit response as a result for each sample and standard of the plate. Magnitude of ECL response is directly proportional to the extent of binding antibody in the test article. All calculations are performed within Excel and the GraphPad Prism software, version 7.0. Readouts are provided as AUC.

## Supplementary Material

20210701-1

## Supplementary Materials

science.sciencemag.org/content/373/6556/eabh1766/suppl/DC1
Figs. S1 to S10 Tables S1 to S3 MDAR Reproducibility Checklist

## Appendix

View/request a protocol for this paper from *Bio-protocol*.
